# Digital engagement and knowledge about zoonoses among dog and cat owners in Rio de Janeiro: a cross-sectional study

**DOI:** 10.3389/fdgth.2026.1746563

**Published:** 2026-02-04

**Authors:** Juliana Lopes de Castro, Liliane de Fátima Antonio Oliveira, Mirian Miranda Cohen, Vitória de Freitas da Silva, Maria Inês Fernandes Pimentel, Daniela Ribeiro Monteiro, Aline Fagundes, Shanna Araujo dos Santos, Rodrigo Caldas Menezes, Sandro Antonio Pereira, Andreza Pain Marcelino

**Affiliations:** 1 Clinical Research and Surveillance Laboratory for Leishmaniasis, Evandro Chagas National Institute of Infectious Diseases, Oswaldo Cruz Foundation, Rio de Janeiro, Brazil; 2 Clinical Epidemiology Laboratory, Evandro Chagas National Institute of Infectious Diseases, Oswaldo Cruz Foundation, Rio de Janeiro, Brazil; 3 Center for Strategic Studies (CEE), Presidency, Oswaldo Cruz Foundation, Rio de Janeiro, Brazil; 4 IBMEC University Center, Rio de Janeiro, Brazil; 5 Laboratory of Clinical Research on Dermatozoonoses in Domestic Animals, Evandro Chagas National Institute of Infectious Diseases, Oswaldo Cruz Foundation, Rio de Janeiro, Brazil

**Keywords:** App, digital health, pets, one health, zoonoses

## Abstract

**Background:**

The One Health approach highlights the interconnectedness between human, animal, and environmental health, reinforcing the importance of public awareness in preventing zoonoses, which remain major sources of emerging infectious diseases. Given the increasing popularity of pet ownership and the widespread use of mobile phones for accessing information, understanding how pet owners engage with digital health content is essential for guiding effective educational strategies. This study assessed the profile of dog and cat owners in Rio de Janeiro, Brazil; their knowledge about zoonoses; patterns of internet and mobile app use; and their interest in a health education app.

**Methods:**

A cross-sectional study was conducted with 166 pet owners recruited from a public veterinary clinic and a private service. Data were collected using a REDCap-based questionnaire administered through face-to-face interviews and online surveys. Descriptive statistics and association tests were performed to explore relationships between demographic variables, digital engagement, and knowledge of zoonoses.

**Results:**

Most respondents were women (78.3%), aged 18–34 years (51.2%) and had higher education degrees (54.2%). Mobile phones were the primary means of internet access. A total of 85.5% had heard of zoonoses, with rabies (86.7%) and sporotrichosis (35.5%) being the most frequently cited. Although 62.6% reported visiting veterinarians twice or more per year, 18.7% did not seek veterinary care. Only 19.9% used pet-care apps, while 74.7% searched online for animal health information. No significant associations were found between demographic characteristics and zoonosis knowledge, predominant mobile phone use, or app use. Significant associations were observed only between mobile phone users and social media access, information-seeking behaviors, knowledge of zoonoses, and pet-care app use.

**Conclusion:**

Mobile phones are the main gateway to health information, indicating strong potential for digital tools to enhance health education and support responsible pet ownership, while respecting veterinarians’ exclusive role in diagnosis and treatment.

## 1 Introduction

In the One Health (OH) framework, human, animal, and environmental health are intrinsically interrelated. According to the World Health Organization (WHO), integrating these three pillars facilitates the entire spectrum of disease control from prevention and diagnosis to response, and contributes to global health security (1). The population represents a critical link within this ecosystem, and the OH approach enhances public understanding of health benefits, risks, and opportunities (2).

Zoonoses are diseases transmitted from animals to humans through bites, vectors, or ingestion of contaminated food. Approximately 60.3% of emerging infectious diseases are zoonotic, therefore, raising public awareness of these risks is an essential component of the health-disease continuum (3).

In recent decades, there has been growing interest in the ownership and care of companion animals. The domestic pet population has increased substantially in the 21st century. In Brazil, it is estimated that eight out of ten households have at least one pet (4). Despite the social and economic benefits of pet ownership, a widespread lack of knowledge persists regarding the potential health risks associated with close human-animal interactions. This increased exposure is particularly concerning for high-risk groups, including children, the elderly, pregnant women, and immunocompromised individuals. Studies conducted by Oliveira-Neto et al. (5) showed that most pet owners are unaware of the modes of transmission, contagion, prevention, and treatment of zoonotic diseases, which exacerbates the risk of dissemination of emerging and re-emerging infections.

Concurrently with the rise in pet ownership, there has been a significant increase in public access to digital communication platforms. Approximately 85% of the Brazilian population has internet access at home, primarily through mobile phones, tablets, and computers (6). Moreover, the use of mobile computing and telecommunications for information seeking has become increasingly prevalent. Mobile phones have been at the forefront of consumer electronics innovation for nearly two decades and currently serve as the primary tool for remote work and education for a large portion of the population (7, 8).

The widespread availability of mobile devices allows for the rapid dissemination of information to the public. However, a pressing need remains for reliable, evidence-based sources to ensure effective education (9). Approximately 80.4% of Brazilian households use mobile phones information as their primary means of internet access (6). Consequently, educational mobile applications (apps) in the health domain can be effectively integrated into individuals’ daily lives.

This study aimed to assess the profile of dog and cat owners in Brazil, their level of knowledge regarding zoonotic diseases, their relationship with the internet and mobile app use, and their interest and expectations concerning the development of a mobile app focused on public health education in the Rio de Janeiro Metropolitan Region.

## 2 Materials and methods

### 2.1 Study design and population

A cross-sectional, observational, and self-reported study targeting dog and cat owners was conducted in the Rio de Janeiro Metropolitan Region between March and October 2025. The study population included dog and cat owners attending the public referral clinic of the Laboratory of Clinical Research on Dermatozoonoses in Domestic Animals, Evandro Chagas National Institute of Infectious Diseases, Oswaldo Cruz Foundation (Group 1), and dog and cat owners receiving private veterinary care (Group 2). The intentional selection of a public referral clinic and a private veterinary service aimed to capture two distinct and common profiles of pet owners with different patterns of access to veterinary care.

### 2.2 Selection criteria

The inclusion criteria comprised dog and/or cat owners aged ≥18 years, residing in Rio de Janeiro, Brazil, who voluntarily agreed to participate and provided written informed consent. Participants were required to be current pet owners at the time of the study. The exclusion criteria included individuals without access to a mobile phone or those unable to complete the electronic questionnaire due to technical or literacy limitations.

### 2.3 Ethical consideration

The project was submitted for review and approved by the Research Ethics Committee (CEP) of the Evandro Chagas National Institute of Infectious Diseases (INI/Fiocruz) (license number 737963, February 13, 2025). As the study did not involve any animal experimentation or manipulation, approval from the Ethics Committee on the Use of Laboratory Animals (CEUA) was not required for this study. The informed consent form was sent to each participant via email via REDCap and was digitally signed before they participation in the study.

### 2.4 Data collection

A semi-structured questionnaire was developed in REDCap and administered through two modalities: face-to-face interviews using tablets, conducted prior to veterinary consultations at the public referral clinic (Group 1), and online (email-based questionnaires) sent by the veterinarian to previously known pet owners receiving private veterinary care (Group 2). A pilot test with three respondents was conducted to assess the feasibility, clarity, and reliability of the instrument, ensuring its appropriateness for collecting data relevant to the study's specific objectives.

The questionnaire included the following categories of information: demographic data (gender, age group, education level, and residential area); internet access (main access device and primary purpose of use); mobile app usage (features that attract user interest and frequency of app use); pet ownership profile (ownership of dogs, cats, or both, and frequency of veterinary visits); prior owners' knowledge (zoonoses awareness) and precise knowledge (zoonoses accurate knowledge); and use of pet-care apps (previous experience with such apps, perceived usefulness, and preferred content or functionalities for an educational pet health app) (Table 1, Supplementary Material).

**Table 1:** Summary of the questionnaire.

Domain	Item number	Variable/content	Question type	Response options	Scoring/coding
Sociodemographic characteristics	1	Gender identity	Multiple choice (single)	Female; Male; Transgender; Non-binary; Other; Prefer not to say	Descriptive
Sociodemographic characteristics	2	Age range	Multiple choice (single)	18–24; 25–34; 35–44; 45–54; 55–64; ≥65 years	Descriptive
Sociodemographic characteristics	3	Education level	Multiple choice (single)	Elementary school; High school; Higher education	Descriptive
Sociodemographic characteristics	4	Full address	Open-ended	Free text	Descriptive
Internet access and digital behavior	5	Internet access device	Multiple choice (multiple)	Mobile phone; Computer; Tablet	Descriptive
Internet access and digital behavior	6	Purpose of internet use	Multiple choice (multiple)	Games; Websites; social media	Descriptive
Internet access and digital behavior	7	App attractiveness factors	Open-ended	Free text	Qualitative analysis
Pet ownership and care	8	Online search for veterinary information	Binary	Yes/No	Descriptive
Pet ownership and care	9	Pet ownership	Multiple choice (single)	Dog; Cat; Both	Descriptive
Pet ownership and care	10	Frequency of veterinary visits	Multiple choice (single)	None; Once a year; Twice or more per year	Descriptive
Zoonoses awareness	11	Awareness of the term zoonoses	Binary	Yes/No	Yes = 1; No = 0
Zoonoses awareness	12	Recognition of zoonotic diseases	Multiple choice (multiple)	Leptospirosis; Rabies; Sporotrichosis; Histoplasmosis; Toxoplasmosis; Sarcoptic mange	1 point per disease (0–6)
Zoonoses accurate knowledge	13	Other zoonoses mentioned	Open-ended	Free text	Qualitative support
Use of digital health tools	14	Previous use of pet-care apps	Binary	Yes/No	Descriptive
Perception of educational apps	15	Interest in zoonosis/pet disease app	Binary	Yes/No	Descriptive
Expectations regarding apps	16	Expected app content	Open-ended	Free text	Qualitative analysis

Zoonoses awareness score range: 0–6 points. Low: 1–2, Moderate: 3–4, High: 5–6 (Item 12).

Zoonoses awareness was assessed using Item 11, which evaluated participants who reported prior familiarity with the term “zoonoses” and Item 12, which required participants to correctly identify specific zoonotic diseases. For Item 12, a scoring system was used to classify knowledge levels as low, moderate, or high, enabling the assessment of zoonoses accurate knowledge. This classification was based on the number of zoonotic diseases the participant reported having heard of and allows for clearer interpretation. Item 13, an open-ended question, provided additional information on the quality and consistency of participants’ conceptual understanding. The responses were categorized into the following groups:

- participants who correctly recognized zoonoses and cited only zoonotic diseases;
- participants who reported knowing zoonoses but cited a mixture of zoonotic and non-zoonotic diseases;
- participants who reported knowing zoonoses but cited only non-zoonotic diseases; and
- participants who reported knowing zoonoses but did not cite any diseases.

### 2.5 Sampling design

Due to practical constraints, a convenience sampling strategy was employed to select the participants. The sample size was calculated considering a finite population of (*N* = 322) individuals. A 95% confidence level (*Z* = 1.96) and a 5% margin of error (*e* = 0.05) were adopted. Since there was no prior estimate of the proportion of the phenomenon of interest, (*p* = 0.5) was used to maximize variability and ensure a conservative sample size. Thus, the minimum required sample to ensure statistical representativeness was 176 participants. The calculation was performed using R software (R Core Team, 2024). The minimum sample size was calculated using the finite population formula:

$$n=\frac{N\cdot Z^{2}\cdot p\cdot (1-p)}{\left(N-1)\cdot e^{2}+Z^{2}\cdot p\cdot (1-p\right)}$$

### 2.6 Data management and analysis

The analysis was carried out using the Statistical Package for the Social Sciences (SPSS) version 16 (SPSS, Inc.,Chicago, IL, USA). Descriptive statistics were performed for categorical variables and presented as frequency tables. To analyse the association of Mobile Phone Access, Knowledge about zoonoses, and Use of pet-care apps with socio-demographic characteristics and other variables, chi-square or Fisher's exact tests were used. A *p*-value <0.05 at a 95% confidence interval was considered statistically significant.

## 3 Results

### 3.1 Socio-demographic and internet access characteristics of the participants

A total of 166 dog and/or cat owners participated in the study. Most participants were women (130, 78.3%) aged between 18 and 34 years (85, 51.2%), and had completed higher education (90, 54.2%). Regarding pet ownership, 61 (36.8%) participants owned dogs, 45 (27.1%) owned cats, and 60 (36.1%) owned both dogs and cats. Among the participants, 84 (50.6%) were interviewed face-to-face (Group 1) and 82 (49.4%) completed the questionnaire by email (Group 2). No statistically significant differences were observed between these two groups regarding the variables analyzed.

One of the survey questions asked, “Where do you most often access the internet today? (More than one option could be selected),” with response options including mobile phone, computer, and tablet. Most respondents reported accessing the internet primarily through mobile phones. Furthermore, no significant associations were found between predominant mobile phone internet use and demographic characteristics, including gender, age, and region of residence (Table 2).

**Table 2:** Demographic characteristics of the participants according to mobile phone access.

Variable	Category	*n* (%)	Mobile Phone Access				*p*-value
			Yes *n* (%)	IC95%	No *n* (%)	IC95%	
Gender	Female	130 (78.3)	124 (79.0)	[72–85]	6 (66.7)	[30–93]	0.409^a^
	Male	36 (21.7)	33 (21.0)	[15–28]	3 (33.3)	[7–70]	
Age group (years)	18–34	85 (51.2)	80 (51.0)	[43–59]	5 (55.6)	[21–86]	0.436^b^
	35–54	46 (27.7)	45 (28.7)	[22–36]	1 (11.1)	[0–48]	
	≥55	35 (21.1)	32 (20.3)	[14–28]	3 (33.3)	[7–70]	
Education level	Primary	5 (3.0)	5 (3.2)	[1–7]	0 (0)	[0–34]	**
	Secondary	71 (42.8)	68 (43.3)	[35–51]	3 (33.3)	[7–70]	
	Tertiary	90 (54.2)	84 (53.5)	[45–61]	6 (66.7)	[30–93]	
Region of residence	North Zone	63 (38.0)	59 (37.6)	[30–46]	4 (44.4)	[14–79]	**
	South Zone	18 (10.8)	16 (10.2)	[6–16]	2 (22.2)	[3–60]	
	West Zone	52 (31.3)	49 (31.2)	[24–39]	3 (33.3)	[7–70]	
	Metropolitan Region*	33 (19.9)	33 (21.0)	[15–28]	0 (0)	[0–34]	
Type of approach	Face-to-face (Group 1)	82 (49.4)	79 (50.3)	[42–58]	3 (33.3)	[7–70]	0.496^a^
	Online (Group 2)	84 (50.6)	78 (49.7)	[42–58]	6 (66.7)	[30–93]	
Type of pet owner	Dog	61 (36.8)	56 (35.7)	[28–44]	5 (55.6)	[21–86]	0.470^b^
	Cat	45 (27.1)	43 (27.4)	[21–35]	2 (22.2)	[3–60]	
	Dog and Cat	60 (36.1)	58 (36.9)	[29–45]	2 (22.2)	[3–60]	
Veterinary visits	None	31 (18.7)	31 (19.7)	[14–27]	0 (0)	[0–34]	**
	Once a year	31 (18.7)	31 (19.7)	[14–27]	0 (0)	[0–34]	
	≥2 per year	104 (62.6)	95 (60.5)	[52–68]	9 (100)	[66–100]	

a Fisher's exact test.

b Pearson Chi-square Test.

* Other municipalities within the Rio de Janeiro Metropolitan Region.

** *p*-value could not be calculated due to zero values in the variable.

Regarding the variables related to internet use purposes (social media, games, and websites) and information-seeking behavior (searching for diseases, veterinarians, and pet treatments), a significant association was observed only between mobile phone users and social media access, whereas no significant differences were found for the other variables analyzed (Table 3).

**Table 3:** Digital behavior characteristics of the participants according to mobile phone access.

Variable	Category	*n* (%)	Mobile Phone Access				*p*-Value
			Yes *n* (%)	IC95%	No *n* (%)	IC95%	
Access to games	Yes	23 (13.9)	23 (14.6)	[10–21]	0 (0)	[0–34]	**
	No	143 (86.1)	134 (85.4)	[79–90]	9 (100)	[66–100]	
Access to websites	Yes	101 (60.8)	93 (59.2)	[51–67]	8 (88.9)	[52–100]	0.091^a^
	No	65 (39.2)	64 (40.8)	[33–49]	1 (11.1)	[0–48]	
Access to social media	Yes	145 (87.3)	140 (89.2)	[83–94]	5 (55.6)	[21–86]	0.016^a^
	No	21 (12.7)	17 (10.8)	[6–17]	4 (44.4)	[14–79]	
Information-seeking behavior*	Yes	124 (76.1)	119 (76.8)	[68–82]	5 (62.5)	[21–86]	0.398^a^
	No	39 (23.9)	36 (23.2)	[17–30]	3 (37.5)	[7–70]	
Interest in apps	Yes	161 (97.0)	153 (97.5)	[94–99]	8 (88.9)	[52–100]	0.246^a^
	No	5 (3.0)	4 (2.5)	[0–6]	1 (11.5)	[0–48]	

a Fisher's exact test.

* Three missing data.

** *p*-value could not be calculated due to zero values in the variable.

**Figure 1:**
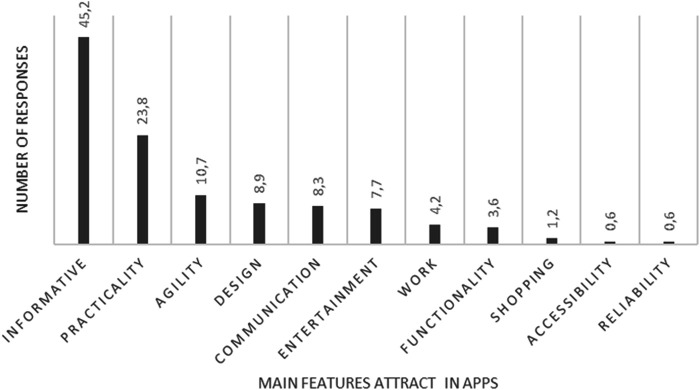
Percentage distribution of Dog and Cat owners’ perceptions of apps available on Mobile phones, tablets, and computers.

When asked what features attract their attention in apps for mobile phones, tablets, or computers, only six participants did not respond directly to the question: one did not provide any answer, one reported not being a user, one did not have internet access, two stated they used videos as their primary source of information, and one used a mobile phone exclusively for making calls. The majority of respondents, however, indicated that the most appealing aspects of apps are the availability of information and their practicality (Figure 1).

### 3.2 Assessment of knowledge of pet owners about zoonoses

A total of 142 (85.5%) of the participants reported having heard of zoonoses (zoonoses awareness). The proportion of the prior knowledge of participants about zoonoses was higher among owners of both dogs and cats than among those who owned only one species (Table 4).

**Table 4:** Demographic characteristics of the participants and dog and cat owners’ knowledge about Zoonoses.

Variable	Category	*n* (%)	Zoonoses awareness				*p*-Value
			Yes *n* (%)	IC95%	No *n* (%)	IC95%	
Gender	Female	130 (78.3)	114 (80.3)	[73–86]	16 (66.7)	[45–84]	0.134^a^
	Male	36 (21.7)	28 (19.7)	[14–27]	8 (33.3)	[16–55]	
Age group (years)	18–34	85 (51.2)	72 (50.7)	[42–59]	13 (54.2)	[33–74]	0.341^a^
	35–54	46 (27.7)	42 (29.6)	[22–38]	4 (16.7)	[5–37]	
	≥55	35 (21.1)	28 (19.7)	[14–27]	7 (29.2)	[13–51]	
Education level	Primary	5 (3.0)	3 (2.1)	[0–6]	2 (8.3)	[1–27]	0.085^a^
	Secondary	71 (42.8)	58 (40.8)	[33–49]	13 (54.2)	[33–74]	
	Tertiary	90 (54.2)	81 (57.0)	[48–65]	9 (37.5)	[19–59]	
Region of residence	North Zone	63 (38.0)	54 (38.0)	[30–47]	9 (37.5)	[19–59]	0.086^a^
	South Zone	18 (10.8)	14 (9.9)	[55–16]	4 (16.7)	[5–37]	
	West Zone	52 (31.3)	49 (34.5)	[27–43]	2 (12.5)	[1–27]	
	Metropolitan Region*	33 (19.9)	25 (17.6)	[12–25]	8 (33.3)	[16–55]	
Type of approach	Face-to-face (Group 1)	82 (49.4)	69 (48.6)	[40–57]	13 (54.2)	[33–74]	*p*-value
	Online (Group 2)	84 (50.6)	73 (51.4)	[43–60]	11 (45.8)	[26–67]	
Type of pet owner	Dog	61 (36.8)	48 (33.8)	[26–42]	13 (54.2)	[33–74]	0.029^a^
	Cat	45 (27.1)	37 (26.1)	[19–34]	8 (33.3)	[16–55]	
	Dog and Cat	60 (36.1)	57 (40.1)	[32–49]	3 (12.5)	[3–32]	
Veterinary visits	None	31 (18.7)	23 (16.2)	[11–23]	8 (33.3)	[16–55]	0.135^a^
	Once a year	31 (18.7)	27 (19.0)	[13–26]	4 (16.7)	[5–37]	
	≥2 per year	104 (62.6)	92 (64.8)	[56–73]	12 (30.0)	[29–71]	

a Pearson Chi-square Test.

* Other municipalities within the Rio de Janeiro Metropolitan Region.

Of the 24 participants (14.5%) who stated they had not heard of zoonoses, all reported being familiar with at least one of the most common zoonotic diseases transmitted in Rio de Janeiro, such as leptospirosis, sporotrichosis, toxoplasmosis, rabies, histoplasmosis, or sarcoptic mange, when these were mentioned by the interviewer. The final proportion of participants who reported having heard of each zoonosis was as follows: leptospirosis (161, 97.0%), rabies (157, 94.6%), sporotrichosis (123, 74.1%), toxoplasmosis (120, 72.3%), sarcoptic mange (144, 86.7%), and histoplasmosis (59, 35.5%). When classified according to the number of zoonotic diseases recognized, 7.8% of participants demonstrated low, 33.1% moderate and 59.0% high levels of zoonoses awareness.

About zoonoses accurate knowledge, approximately 58 participants (108 missing data) of the 166 participants (34.9%) mentioned being familiar with other diseases. Of these, 22 (13.3%) correctly recognized zoonoses (zoonoses accurate knowledge) and cited only zoonotic diseases, 4 (2.4%) reported knowing zoonoses but cited a mixture of zoonotic and non-zoonotic diseases, 23 (13.9%) reported knowing zoonoses but cited only non-zoonotic diseases, and 9 (5.4%) reported knowing zoonoses but did not cite any diseases. Diseases incorrectly self-reported as zoonoses included canine distemper, parvovirus infection, chronic kidney disease, and oncological conditions.

When comparing the proportions of pet owners who reported having heard about zoonoses, this proportion was higher among individuals who engage in information-seeking behavior than among those with no prior knowledge of zoonoses (Table 5).

**Table 5:** Digital behavior characteristics of the participants according to dog and cat owners’ knowledge about zoonoses.

Variable	Category	*n* (%)	Knowledge about Zoonoses				*p*-Value
			Yes *n* (%)	IC95%	No *n* (%)	IC95%	
Access to games	Yes	23 (13.9)	20 (14.1)	[9–21]	3 (15.5)	[3–32]	1.000^a^
	No	143 (86.1)	122 (85.9)	[79–91]	21 (87.5)	[68–97]	
Access to websites	Yes	101 (60.8)	89 (62.7)	[54–71]	12 (50.0)	[29–71]	0.239^b^
	No	65 (39.2)	53 (37.3)	[29–46]	12 (50.0)	[29–71]	
Access to social media	Yes	145 (87.3)	123 (86.6)	[80–92]	22 (91.7)	[73–99]	0.742^a^
	No	21 (12.7)	19 (13.4)	[8–20]	2 (8.3)	[1–27]	
Information-seeking behavior	Yes	124 (76.1)	114 (82.0)	[73–86]	10 (41.7)	[22–63]	<0,001^b^
	No	39 (23.9)	25 (18.0)	[12–25]	14 (58.3)	[37–78]	
Interest in apps	Yes	161 (97.0)	137 (96.5)	[92–99]	24 (100)	[86–100]	1.000^a^
	No	5 (3.0)	5 (3.5)	[1–8]	0 (0)	[0–14]	

a Fisher Exact Test.

b Pearson Chi-square Test.

### 3.3 Profile of dog and cat owners regarding pet health care practices

Among these owners, 104 (62.6%) reported taking their pets to a veterinarian twice or more per year, 31 (18.7%) reported taking their pets once annually and 31 (18.7%) reported not seeking veterinary care. Overall, 33 participants (19.9%) reported using an app to obtain guidance on pet care, while 133 (80.1%) did not (Table 6).

**Table 6:** Demographic characteristics of participants and their use of pet-care apps.

Variable	Category	*n* (%)	Use of pet-care apps				*p*-Value
			Yes *n* (%)	IC95%	No *n* (%)	IC95%	
Gender	Female	130 (78.3)	30 (90.9)	[76–98]	100 (75.2)	[67–82]	0.050^b^
	Male	36 (21.7)	3 (9.1)	[2–24]	33 (24.8)	[18–33]	
Age group (years)	18–34	85 (51.2)	14 (42.4)	[25–61]	71 (53.4)	[45–62]	0.104^b^
	35–54	46 (27.7)	14 (42.4)	[25–61]	32 (24.1)	[17–32]	
	≥55	35 (21.1)	5 (15.2)	[5–32]	30 (22.6)	[16–31]	
Education level	Primary	5 (3.0)	1 (3.0)	[0–16]	4 (3.0)	[1–8]	0.465^b^
	Secondary	71 (42.8)	11 (33.3)	[18–52]	60 (45.1)	[36–54]	
	Tertiary	90 (54.2)	21 (63.6)	[45–80]	69 (51.9)	[43–61]	
Region of residence	North Zone	63 (38.0)	11 (33.3)	[18–52]	52 (39.1)	[31–48]	0.427^b^
	South Zone	18 (10.8)	2 (6.1)	[0–20]	16 (12.0)	[8–19]	
	West Zone	52 (31.3)	14 (42.4)	[25–61]	38 (28.6)	[21–37]	
	Metropolitan Region*	33 (19.9)	6 (18.2)	[7–35]	27 (20.3)	[14–28]	
Type of approach	Face-to-face (Group 1)	82 (49.4)	15 (45.5)	[28–64]	67 (50.4)	[42–59]	0.613^b^
	Online (Group 2)	84 (50.6)	18 (54.5)	[36–72]	66 (49.6)	[41–58]	
Type of pet owner	Dog	61 (36.8)	7 (21.2)	[9–39]	54 (40.6)	[32–49]	0.067^b^
	Cat	45 (27.1)	9 (27.3)	[13–46]	36 (27.1)	[20–35]	
	Dog and Cat	60 (36.1)	17 (51.5)	[34–69]	43 (32.3)	[24–41]	
Veterinary visits	None	31 (18.7)	3 (9.1)	[2–24]	28 (21.1)	[14–29]	0.081^b^
	Once a year	31 (18.7)	10 (30.3)	[16–49]	21 (15.8)	[10–23]	
	≥2 per year	104 (62.6)	20 (60.6)	[42–77]	84 (63.2)	[54–71]	

a Fisher Exact Test.

b Pearson Chi-square Test.

* Other municipalities within the Rio de Janeiro Metropolitan Region.

In addition, most participants (124, 76.1%) reported using the internet to search for information on animal diseases, veterinarians, or treatment options, whereas 39 (23.9%) stated that they did not perform such research and, 3 (1.8%) of the participants did not respond (missing data). The proportion of participants reporting knowledge about zoonoses was higher among those who demonstrated information-seeking behavior (Table 7).

**Table 7:** Digital behavior characteristics of the participants and their use of pet-care apps.

Variable	Category	*n* (%)	Use of pet-care apps				*p*-Value
			Yes *n* (%)	IC95%	No *n* (%)	IC95%	
Access to games	Yes	23 (13.9)	5 (15.2)	[5–32]	18 (13.5)	[8–21]	0.782^a^
	No	143 (86.1)	28 (84.8)	[68–95]	115 (86.5)	[79–92]	
Access to websites	Yes	101 (60.8)	23 (69.7)	[51–84]	78 (58.6)	[50–67]	0.244^b^
	No	65 (39.2)	10 (30.3)	[16–49]	55 (41.4)	[33–50]	
Access to social media	Yes	145 (87.3)	29 (87.9)	[72–97]	116 (87.8)	[80–92]	1.000^a^
	No	21 (12.7)	4 (12.1)	[3–28]	17 (12.8)	[8–20]	
Information-seeking behavior	Yes	124 (76.1)	32 (97.0)	[84–100]	92 (70.8)	[61–77]	0.002^b^
	No	39 (23.9)	1 (3.0)	[0–16]	38 (29.2)	[21–37]	
Interest in apps	Yes	161 (97.0)	32 (97.0)	[84–100]	129 (97.0)	[92–99]	1.000^a^
	No	5 (3.0)	1 (3.0)	[0–16]	4 (3.0)	[1–8]	

a Fisher Exact Test.

b Pearson Chi-square Test.

**Figure 2:**
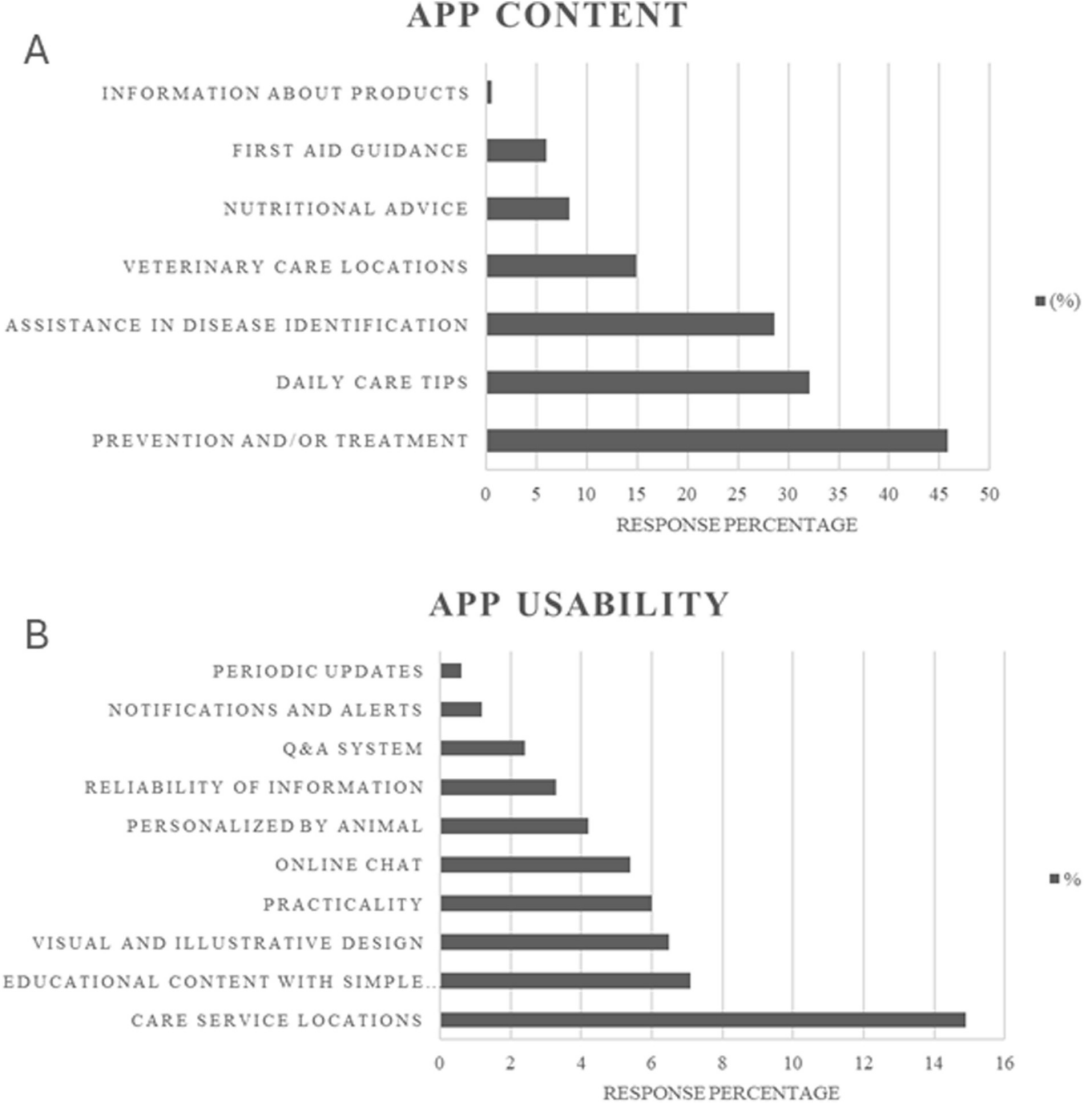
Distribution of dog and cat owners’ responses regarding desired features for a pet-care mobile application. Preferences related to app functionalities **(A)** preferences related to app usability **(B)**.

The majority of pet owners (97%) considered the development of an app providing information on the main clinical signs and symptoms of diseases in pets to be relevant and useful. Participants also suggested additional topics and functionalities they would like to see included in an app focused on veterinary health education and guidance. Responses were grouped into categories of app content and usability (Figure 2).

## 4 Discussion

One of the main focuses of the One Health approach is the transmission of infectious diseases among wildlife, domestic animals, including companion animals, and humans. The human and veterinary medical communities must work collaboratively on issues that impact the health and well-being of both humans and animals (10). Small companion animals live in close association with human households in most cultures and countries worldwide, highlighting their relevance in the context of zoonotic disease prevention and control (11, 12).

Considering the descriptive nature of the study and the sample obtained, the analyses were used for exploratory purposes, without assuming causality. Our study employed two different strategies for questionnaire application (Groups 1 and 2), and no significant differences were observed between the two groups in the response profiles for the outcomes assessed. We included owners of dogs, cats, and both in our study to ensure that the profile assessment encompassed differences related to owning multiple pets of different species. Our results are consistent with findings in the literature, which also report a higher proportion of female pet owners (13, 14). These demographic findings are consistent with data from the National Union of the Animal Health Products Industry (Sindan), which identified women as the main pet owners in Brazil (15). Regarding age, a previous study conducted in Texas found that individuals aged 24–44 were more likely to own pets, a pattern that aligns with the age distribution observed in our study, where the 18–34-year age group also showed the highest proportion (16).

Our results are consistent with epidemiological studies reporting weak or non-statistically significant associations between age, sex, and educational attainment and the outcomes assessed, suggesting that other determinants may be involved (17). The study results indicate that pet owners are generally familiar with the internet, primarily using it for social media, reflecting a trend observed worldwide. Technological advancements have diversified online platforms, enabling interactions through professional websites, discussion forums, multimedia-sharing networks, and other channels (18). In our study, the proportion of individuals who accessed social media was higher among those who primarily accessed the internet via mobile phones, underscoring the central role of mobile devices in digital engagement. Despite the variety of available apps and their different functions, our results show that most users are motivated to use apps primarily for their practicality and informative content, whereas communication and entertainment were cited by only 8.3% and 7.7% of participants, respectively. These findings suggest that mobile applications have strong potential to disseminate knowledge and promote behaviors that support public health initiatives.

In terms of pet care, although the majority of owners reported taking their pets to the veterinarian more than twice a year, approximately 19% indicated that they did not seek veterinary care at all. This concerning finding suggests that some individuals may be unaware of the importance of preventive measures, such as vaccination and routine health monitoring, prior to disease onset (19), which is consistent with Park et al. (20) that approximately 20% of dog owners do not seek veterinary care annually in the United States.

Although most participants reported using the internet to search for information about animal diseases, veterinarians, or treatment options, the proportion of participants seeking to use pet-care apps was low, possibly due to low accessibility or limited knowledge and availability of apps specifically developed to provide reliable and educational content about animal health. However, when comparing the use or non-use of pet-care apps in relation to information-seeking behavior on animal diseases, veterinarians, or treatment options, a significant association was observed. Furthermore, most participants expressed interest in the development of an app that provides information on the main signs and symptoms of diseases in pets, highlighting the potential demand for such tools.

A substantial proportion of pet owners reported having heard of zoonoses. Although zoonoses awareness was high (85.5%), zoonoses accurate knowledge was low, conceptual understanding was heterogeneous and inconsistent, reflected by the incorrect classification of non-zoonotic conditions such as parvovirus, chronic kidney disease, and cancer. A study conducted in Portugal (20) found that 73.6% of participants considered animals as potential sources of human diseases, yet only 25.9% reported knowing the definition of a zoonosis. This highlights the need to strengthen educational communication among veterinarians, physicians, pet owners, and the general public to reduce the risk of acquiring and transmitting such diseases. Our results indicate that participants who owned both dogs and cats were more likely to report knowledge about zoonoses than those who owned only one species. A possible explanation for this result may be the pet owner's broader experience with animal care, more frequent veterinary visits, and/or adoption of preventive measures necessary for different species. Furthermore, owners of multiple species may demonstrate a greater interest in animal health and welfare, which could contribute to greater awareness of zoonotic risks. Similar trends have been reported in previous studies, which suggest that higher levels of involvement and interaction with animals are associated with better knowledge and preventive practices regarding zoonoses (5–20).

Participants highlighted two main aspects as essential for the app's effectiveness: usability and educational content. Practicality, intuitive navigation, and attractive visual features were considered fundamental for user engagement, while the inclusion of functionalities to assist in locating veterinary professionals reflected the demand for easy access to specialized guidance. These preferences align with studies demonstrating that usability, aesthetics, and user-centered design increase adoption of health apps (21). Regarding content, participants valued information on disease identification, general pet care, and disease prevention and treatment, highlighting the app's educational potential. However, the diagnosis and treatment of animal diseases are the exclusive responsibility of veterinarians, and the app's content should remain educational and not prescriptive. Thus, apps that combine reliable information with an attractive and user-friendly design while respecting the limits of veterinary professional practice can promote engagement and support animal and public health.

Among the limitations of this study, it should be noted that the calculated sample size of 176 participants was not reached. Therefore, the results should not be extrapolated to the general population of pet owners in Rio de Janeiro but rather interpreted within the context of the specific veterinary care settings investigated. In addition, the low number of events per variable in the groups prevented an exploratory analysis using logistic regression. Another limitation is that the questionnaire applied did not allow for the presentation of the frequency of use of pet care apps and examples of apps used. Future research involving pet owners recruited from a wider range of veterinary services would be valuable to further elucidate aspects that could not be fully addressed in the present study.

## 5 Conclusion

Mobile phones are the primary means of accessing applications, with information on prevention and treatment being the most frequently searched topics. User searches for specific diseases were associated with self-reported knowledge and the use of applications for pet care. Our study highlights the difference between self-reported knowledge about zoonoses and the participants’ actual knowledge, which was substantially lower. Strategies for raising awareness and improving actual knowledge about zoonoses are needed, and digital tools through educational mobile applications can be a strategy supported by a One Health approach. Importantly, any guidance on disease management should remain educational, respecting the exclusive role of veterinarians in diagnosis and treatment.

## Data Availability

The raw data supporting the conclusions of this article will be made available by the authors, without undue reservation.
