# Supplementary material for: Digital engagement and knowledge about zoonoses among dog and cat owners in Rio de Janeiro: a cross-sectional study
Source: Front Digit Health. 2026 Feb 4;8:1746563. doi: 10.3389/fdgth.2026.1746563 (PMC12913369; doi:10.3389/fdgth.2026.1746563)
Supplement: Supplementary file 1 [file Datasheet1.docx]

Supplementary Material

**Questionnaire**

1. What is your gender identity?

- Female
- Male
- Transgender
- Non-binary
- Other
- Prefer not to say

2. What is your age range?

- 18–24 years
- 25–34 years
- 35–44 years
- 45–54 years
- 55–64 years
- 65 or older

3. Education level:

- Elementary school
- High school
- Higher education

4. Full address: _____________________________________________________ (Open field)

5. How do you mostly access the internet today? (Multiple options may be selected)

- Mobile phone
- Computer
- Tablet

6. For what purposes do you access the internet today? (Multiple options may be selected)

- Games
- Websites
- Social media

7. What attracts you most to apps for phones, tablets, and computers?

_________________________________________________________(Open field)

8. Do you search online for diseases, veterinarians, or animal diseases treatments?

- Yes
- No

9. Do you have a dog or cat?

- Dog
- Cat
- Both

10. If yes, how many times a year do you take your pet to the vet?

- None
- Once
- Twice or more a year

11. Have you heard of zoonoses (diseases transmitted from animals to humans)?

- Yes
- No

12. Which of these diseases have you heard of?

- Leptospirosis
- Rabies
- Sporotrichosis
- Histoplasmosis
- Toxoplasmosis
- Sarcoptic mange (scabies)

13. If you've heard of other diseases, which ones?

__________________________________________________(Open field)

14. Have you ever used an app to guide you on pet care?

- Yes
- No

15. Do you think it's interesting to create an app that informs the main signs and symptoms of pet diseases?

- Yes
- No

16. What do you expect to find in an app about veterinary care/guidance?

__________________________________________________________________ (Open field
